# Draft Genome Sequence of *Fusobacterium nucleatum* subsp. *vincentii* ChDC F8, Isolated from a Human Subgingival Plaque in the Republic of Korea

**DOI:** 10.1128/genomeA.01040-13

**Published:** 2013-12-12

**Authors:** Soon-Nang Park, Eugene Cho, Hwa-Sook Kim, Dae-Soo Kim, Jaeeun Jung, Jeong-Hun Baek, Yun Kyong Lim, Eojin Jo, Young-Hyo Chang, Jeong Hwan Shin, Jihun Kim, Sang-Haeng Choi, Jihee Kang, YongUn Choi, Hong-Seog Park, Hongik Kim, Joong-Ki Kook

**Affiliations:** Korean Collection for Oral Microbiology and Department of Oral Biochemistry, School of Dentistry, Chosun University, Gwangju, Republic of Koreaa; Department of Dental Hygiene, Chunnam Techno University, Chunnam, Republic of Koreab; Human Derived Material Center, Korea Research Institute of Bioscience and Biotechnology (KRIBB), Daejeon, Republic of Koreac; Macrogen, Inc., Gasan-dong, Seoul, Republic of Koread; Korean Collection for Type Cultures, Biological Resource Center, KRIBB, Daejeon, Republic of Koreae; Department of Laboratory Medicine, Busan Paik Hospital, Inje University College of Medicine, Busan, Republic of Koreaf; Research Institute of Unitech Science Co., Ltd., Daejeon, Republic of Koreag; R&D Division, Vitabio, Inc., Daejeon, Republic of Koreah

## Abstract

*Fusobacterium nucleatum* is a Gram-negative, nonmotile, obligately anaerobic rod bacterium which might play an important role in the initiation and progression of periodontal diseases. *F. nucleatum* subsp. *vincentii* ChDC F8 (KCOM 1231) was isolated from a human gingivitis lesion. Here, we report the draft genome sequence of the strain.

## GENOME ANNOUNCEMENT

*Fusobacterium nucleatum* is a Gram-negative, nonmotile, obligately anaerobic rod bacterium which might play an important role in the initiation and progression of periodontal diseases (1, 2). *F. nucleatum* is classified into five subspecies: *nucleatum*, *polymorphum*, *vincentii*, *animalis*, and *fusiforme* (3, 4). Recently, *F. nucleatum* subsp. *vincentii* and *F. nucleatum* subsp. *fusiforme* were classified as a single subspecies, *F. nucleatum* subsp. *vincentii*, by phylogenetic analysis using a single sequence (24,715 bp) of 22 concatenated housekeeping genes (5). In this report, we present the draft genome sequence of *F. nucleatum* subsp. *vincentii* ChDC F8 (KCOM 1231), which was isolated from a human gingivitis lesion (6).

Draft sequencing was performed by the Macrogen Co., (Seoul, South Korea) using the Illumina Hiseq 2000 system sequencing technology. We constructed 101 paired-end sequencing libraries with insert sizes of about 200 bp and generated 54,550,386 bp of usable sequence. We assembled the reads using SOAPdenovo (http://soap.genomics.org.cn). SOAPdenovo v. 1.05 was run with option K79 and configuration options reverse_seq of 0 (standard mate-pair orientation), asm_flags of 3 (try harder to build large contigs), rank of 1 (reads were used while scaffolding). The reads were assembled into 186 contigs with a size range between 202 and 89,584 bp (total 1,989,265 bp) and the GC content was 26.87%. Open reading frames were predicted and annotated using the Glimmer 3.02 modeling software package (7). Then, the GO classes were grouped into a total of 124 GO-Slim terms using the web tool CateGOrizer (8). The predicted protein sequences were annotated as Gene Ontology by the basic local alignment search tool (BLAST).

The draft genome of *F. nucleatum* subsp. *vincentii* ChDC F8 contained 1,808 protein-coding genes, 1 5S rRNA and 28 tRNA genes, and several key pathways for amino acids, carbohydrates, lipids, and organic acids. Biosynthetic pathways exist for at least four  amino acids, aspartate, asparagine, glutamate, and glutamine. The draft genome sequence contains virulence factors such as hemolysin, multidrug resistance proteins, 5-nitroimidazole antibiotic resistance proteins, beta-lactamase, butyrate fermentation-related genes, zinc metalloprotease, serine protease, outer membrane porin F, toxin YoeB, TonB protein, and TolC. The genome contained oxidative stress-response genes such as glutathione peroxidase, glutaredoxin, NADH oxidase, lactoylglutathione lyase, hydroxyacylglutathione hydrolase, CoA-disulfide reductase, rubrerythrin, nicotinate phosphoribosyltransferase, NAD-dependent protein deacetylase of the SIR2 family, and NAD-dependent glyceraldehyde-3-phosphate dehydrogenase. The genome also contained four sensor kinases and three response regulators of two-component systems.

### Nucleotide sequence accession numbers.

This whole-genome shotgun project has been deposited at DDBJ/EMBL/GenBank under the accession number ATKB00000000. The version described in this paper is the first version, ATKB01000000.
